# Supplementary material for: Effectiveness of cognitive–behavioural therapy: a protocol for an overview of systematic reviews and meta-analyses
Source: BMJ Open. 2018 Dec 14;8(12):e025761. doi: 10.1136/bmjopen-2018-025761 (PMC6303684; doi:10.1136/bmjopen-2018-025761)
Supplement: Supplementary file 1 [file bmjopen-2018-025761supp001.pdf]

Appendix A: Search Strategy for MEDLINE

Database & platform: Ovid MEDLINE(R) Epub Ahead of Print, In-Process & Other Non-Indexed Citations, Ovid MEDLINE(R) Daily and Ovid MEDLINE(R) 1946 to Present

Search strategy development date: 9 March 2018

1. (cognitive adj2 behavio?r adj3 (therap$ or theor$ or intervention$ or train$ or treatment$ or psychotherap$ or programme$ or program$ or method$ or approach$)).ti,ab,kw.

2. (cognitive adj2 behavio?ral adj3 (therap$ or theor$ or intervention$ or train$ or treatment$ or psychotherap$ or programme$ or program$ or method$ or approach$)).ti,ab,kw.

3. CBT.ti,ab,kw.

4. Cognitive Therapy/

5. or/1-4

6. Meta-Analysis as Topic/

7. meta analy$.tw.

8. metaanaly$.tw.

9. Meta-Analysis/

10. (systematic adj (review$1 or overview$1)).tw.

11. exp Review Literature as Topic/

12 or/6-11

13. cochrane.ab.

14. embase.ab.

15. (psychlit or psyclit).ab.

16. (psychinfo or psycinfo).ab.

17. (cinahl or cinhal).ab.

18. science citation index.ab.

19. bids.ab.

20. cancerlit.ab.

21. or/13-20

22. reference list$.ab.

23. bibliograph$.ab.

24. hand-search$.ab.

25. relevant journals.ab.

26. manual search$.ab.

27. or/22-26

28. selection criteria.ab.

29. data extraction.ab.

30. 28 or 29

31. Review/

32. 30 and 31

33. Comment/

34. Letter/

35. Editorial/

36. animal/

37. human/

38. 36 and 37

39. 36 not 38

40. or/33-35,39

41. 12 or 21 or 27 or 32

42. 41 not 40

43. 5 and 42

44. limit 43 to yr="1992-2018"
