# Supplementary material for: Effectiveness of cognitive–behavioural therapy: a protocol for an overview of systematic reviews and meta-analyses
Source: BMJ Open. 2018 Dec 14;8(12):e025761. doi: 10.1136/bmjopen-2018-025761 (PMC6303684; doi:10.1136/bmjopen-2018-025761)
Supplement: Author's manuscript [file bmjopen-2018-025761.draft_revisions.pdf]

BMJ Open is committed to open peer review. As part of this commitment we make the peer review history of every article we publish publicly available.

When an article is published we post the peer reviewers' comments and the authors' responses online. We also post the versions of the paper that were used during peer review. These are the versions that the peer review comments apply to.

The versions of the paper that follow are the versions that were submitted during the peer review process. They are not the versions of record or the final published versions. They should not be cited or distributed as the published version of this manuscript.

BMJ Open is an open access journal and the full, final, typeset and author-corrected version of record of the manuscript is available on our site with no access controls, subscription charges or pay-per-view fees (http://bmjopen.bmj.com).

If you have any questions on BMJ Open's open peer review process please email info.bmjopen@bmj.com

BMJ Open

Effectiveness of Cognitive Behavioural Therapy: A protocol for an overview of systematic reviews and meta-analyses

Journal:	*BMJ Open*
Manuscript ID	bmjopen-2018-025761
Article Type:	Protocol
Date Submitted by the Author:	02-Aug-2018

Complete List of Authors:	Fordham, Beth; University of Oxford Nuffield Department of Orthopaedics Rheumatology and Musculoskeletal Sciences<br>Sugavanam, Thavapriya; University of Oxford Nuffield Department of Orthopaedics Rheumatology and Musculoskeletal Sciences,<br>Hopewell, Sally; University of Oxford, Centre for Statistics in Medicine, Nuffield Department of Orthopaedics, Rheumatology and Musculoskeletal Sciences<br>Hemming, Karla; University of Birmingham, Public Health<br>Howick, Jeremy; University of Oxford, Primary Care Health Sciences<br>Kirtley, Shona; University of Oxford, Centre for Statistics in Medicine<br>dasNair, Roshan; Institute of Mental Health, University of Nottingham<br>Hamer-Hunt, Julia<br>Lamb, Sarah; University of Oxford Nuffield Department of Orthopaedics Rheumatology and Musculoskeletal Sciences

Keywords:	Cognitive behaviour therapy, Overview of systematic reviews, MENTAL HEALTH, PSYCHIATRY

SCHOLARONE™
Manuscripts

Cognitive Behavioural Therapy: A protocol for an overview of systematic reviews and meta-analyses

Beth Fordham [1], Thavapriya Sugavanam [1], Sally Hopewell [2], Karla Hemming [3], Jeremy Howick [4], Shona Kirtley [2], Roshan das Nair [5], Julia Hamer-Hunt [6], and Sarah E Lamb [1]

Corresponding author: Beth Fordham beth.fordham@ndorms.ox.ac.uk [1] Nuffield Department of Orthopaedics, Rheumatology and Musculoskeletal Sciences, University of Oxford, Botnar Research Centre, Windmill Road, Headington, Oxford OX3 7LD

Author affiliations: [2] Centre for Statistics in Medicine, Nuffield Department of Orthopaedics, Rheumatology and Musculoskeletal Sciences, University of Oxford. [3]Public Health, Epidemiology and Biostatistics, Institute of Applied Health Research, University of Birmingham. [4]Department of Primary Care Health Sciences, Centre for Evidence-Based Medicine, University of Oxford, [5]Institute of Mental Health, University of Nottingham, [6]Patient representative.

Email: Thavapriya Sugavanam priya.sugavanam@ndorms.ox.ac.uk, Sally Hopewell sally.hopewell@csm.ox.ac.uk, Karla Hemming K.Hemming@bham.ac.uk, Jeremy Howick jeremy.howick@phc.ox.ac.uk, Shona Kirtley shona.kirtley@csm.ox.ac.uk, Roshan das Nair Roshan.Dasnair@nottingham.ac.uk, Julia Hamer-Hunt julia.hamerhunt@gmail.com and Sallie Lamb sarah.lamb@ndorms.ox.ac.uk.

Authors' contributions: BF is the guarantor. PS developed the protocol with BF. SH provided expertise on design and methodology. KH provided expertise on methodology in particular panoramic meta-analysis. JH provided expertise in methodology. SK developed the draft search strategy plan. RdN, and JHH provided clinical contextual feedback. SL provided expertise in all areas of design, methodology and rigour. All authors read, provided feedback and approved the final manuscript.

Funding statement: This overview of systematic reviews is funded by the National Institute for Health Research, Health Technology Assessment Programme (funding reference number HTA 15/174: Cognitive Behavioural Therapy: An overview of systematic reviews and meta-analyses). The University of Oxford is the sponsor of this overview of systematic reviews. The NIHR HTA programme is funding this overview of systematic reviews. They will provide funding to NDORMS (University of Oxford) who will carry out the research and store the data. The NIHR HTA programme is not involved in any other aspect of the project design, data collection or analyses. They will be made aware of the findings at milestones throughout the project.

Acknowledgments:
Professor Sallie Lamb receives funding from the National Institute for Health Research (NIHR) Collaboration for Leadership in Applied Health Research and Care Oxford at Oxford Health NHS Foundation Trust. The views expressed are those of the author(s) and not necessarily those of the NHS, the NIHR or the Department of Health and Social Care.

Competing Interests statement:
No authors have any competing interests to declare.

Word count: 3557words

For peer review only

Abstract

Introduction: Cognitive behavioural therapy (CBT) is a psychological therapy which has been used to improve patient well-being across multiple mental and physical health problems. Its effectiveness has been examined in thousands of randomised control trials which have been synthesised into hundreds systematic reviews. The aim of this overview is to map, synthesise and assess the reliability of evidence generated from these systematic reviews of the effectiveness of CBT across all health conditions, patient groups and settings.

Methods and analysis: We will run our search strategy, to identify systematic reviews of CBT, within Database of Abstracts of Reviews of Effects, the Cochrane Library of Systematic Reviews, MEDLINE, EMBASE, PsycInfo, CINAHL, Child Development and Adolescent Studies and OpenGrey between January 1992 and 25th April 2018. Independent reviewers will sift, perform data extraction in duplicate and assess the quality of the reviews using the Assessing the Methodological Quality of Systematic Reviews (version.2) tool. The outcomes of interest include: health related quality of life, depression, anxiety and other psychological and physical/physiological outcomes prioritised in the individual reviews. The evidence will be mapped and synthesized where appropriate by health problem, patient sub-groups, intervention type, context and outcome.

Ethics and dissemination: Ethical approval is not required as this is an overview of published systematic reviews. We plan to publish results in peer reviewed journals, present at international and national academic, clinical and patient conferences.

Registration details:

Our protocol was registered with the International Prospective Register of Systematic Reviews (PROSPERO) on 17th April 2018 (registration number: CRD42017078690).

Strengths and limitations of this study

- A strength of this study is that it is the only up to date overview of systematic reviews examining randomised control trials of the effectiveness of Cognitive Behavioural Therapy across all health problems, populations and settings.
- Another strength is that our method allows us to map the available evidence and develop a framework to suggest where evidence can be generalised to and from.
- The main weakness is that we will only include systematic reviews which explicitly state, “Cognitive behavioural therapy” (including all synonyms) in their abstract, title or keywords. This excludes broader reviews which encapsulate the CBT within “psychological interventions.”
- Another weakness is that, we are reliant on the information provided in the systematic reviews therefore we might omit RCTs if they are not included in the reviews we synthesis.

For peer review only

Introduction

The cognitive behavioural model theorises that when living with a physical or mental health problem the way in which we think and behave in response to the problem can influence our emotional and physical well-being and consequently our overall quality of life. The relationships between cognitions, behaviours, emotions and physical responses are all considered bidirectional[1,2].

Cognitive Behavioural Therapy (CBT) is a talking therapy, which targets identifying maladaptive thoughts and behaviours and challenging them, trying to develop different ways of thinking and acting to improve the emotional and physical outcomes for patients. CBT has a core set of competencies which can be applied trans-diagnostically, however it has also been tailored for use in specific populations, such as CBT-Insomnia. Most CBT is delivered in adherence with CBT process manuals specific to the health problem. Roth and Pilling, on behalf of the Department of Health, developed a set of core competencies for CBT and included a division between high and low intensity CBT [3]. They defined high intensity as formal CBT with a CBT-trained health professional predominantly delivered face to face in an individual or group format. Low intensity interventions focus on patient self-help and can be delivered by health professionals with very little to fairly comprehensive CBT training and via several platforms (internet, phone, paper-based). This distinction can become less clear in some forms of CBT, called "blended care", where high intensity therapy is combined with low intensity self-help methods.

The effectiveness of CBT has been evaluated with randomised control trials (RCTs) , which have been synthesised into systematic reviews across numerous physical and mental health problems from schizophrenia[4] to low back pain[5]. We recognised some consistency across the CBT systematic reviews e.g. improving symptoms of insomnia in adults with various health problems [6], [7], [8]. However, we also identified areas with conflicting evidence for example with regards to the efficacy of CBT in reducing relapse in schizophrenia [9,10].

Whilst we are cognisant the volume and variety of available systematic reviews of CBT we are not aware of the quality of the reviews conducted across different health problems, populations and settings. Another limitation of current evidence, is that short term changes to function as a result of CBT do not guarantee long term changes[11,12] and much of the evidence focuses on shorter term outcomes.

This overview will explore the effects of CBT across all health problems, in all populations and in all settings. The primary outcome will be Health Related Quality of Life (HRQL) with the aim of capturing, to some degree, the broader, general, biopsychosocial influence of CBT in addition to its impact upon the specific functional outcomes.

Rationale

Our scoping work suggests there are more than 500 systematic reviews of CBT and there has been no published overview of systematic reviews since 2004. We will address this gap, and aim to map for which populations there are systematic reviews of RCTs examining CBT and document how well these reviews were conducted. Within each population we will identify whether (a) there is a need for new or better quality systematic reviews or RCTs or (b) that CBT worsens/does not alter/improves generic (HRQL) and problem specific health outcomes in comparison to active or not active control conditions in the short or long term follow-up period.

Objectives

The specific objectives include:

(1) Stage one: A Map of the evidence
   a. Map and assess the quality of available evidence
(2) Stage two: A synthesis of the evidence
   a. A descriptive and a panoramic meta-analytic synthesis of the evidence (PMA) by ICD-11 health problem categorisations and by common outcomes (HRQL, depression, anxiety and the most common physical/physiological outcome).

For peer review only - http://bmjopen.bmj.com/site/about/guidelines.xhtml

b. Sub-group analysis to explore high versus low intensity CBT (as defined by Roth and Pilling, 2007[3]) for a health problem.

Methods

Patient and Public Involvement

We are working with a CBT expert consultation group (ECG) consisting of clinical academics (n=7), research academics (n=9) and service users (n=4). We meet with this group face to face twice and communicate via phone/email throughout the overview process to guide our protocol development, synthesis strategy, and interpretation. We hope the ECG will guide our overview to produce clinically meaningful outputs. The group will not be involved in any of the data extraction or quality assessment to ensure no undue influence.

Methods

We shall perform two stages within this overview. Stage one is to identify all the available systematic reviews of CBT, which include RCT evidence then to map the available evidence along with a quality assessment of the included reviews. The second stage will be to meaningfully synthesise the evidence by common outcomes across health problems and to specifically examine the comparative effectiveness of high and low intensity CBT.

Stage one: Mapping the evidence

This stage will detail how we will identify and select the systematic reviews for inclusion in order to generate a comprehensive map of the evidence.

Eligibility criteria

To be included in the evidence map and overview of systematic reviews, studies must meet the following criteria:

Type of studies: We will include systematic reviews of randomised control trials (RCTs) which evaluate the effects of CBT. We will include systematic reviews which include both randomised and non-randomised trials so long as the review has summarised the RCT evidence independently.

To be included, systematic reviews must fulfil a minimum of 4 methodological criteria as defined by the Centre for Reviews and Dissemination (CRD), University of York, as part of the Database of Reviews of Effects (DARE) database (http://www.crd.york.ac.uk/crdweb) [13]: (1) inclusion/exclusion criteria reported; (2) adequate search strategy; (3) included studies synthesised; (4) quality of the included studies assessed; (5) sufficient details about the included studies reported. The University of York have provided us with detailed definitions for each of these criteria. For example the minimum sufficient details of the individual studies would be details of the population, setting, interventions and results for every included study (in text, tables or online appendices).

Type of participants: We will include systematic reviews of RCTs, which include data from all age groups and any gender. We will include all health problems recognised within the ICD-11.

Setting: We will include systematic reviews of RCTs that have been conducted in any context or country.

Intervention: We will only include systematic reviews where Cognitive Behavioural Therapy (or other CBT synonyms) has been explicitly reported in the review title, abstract or key words. We will include all formats of CBT. We will classify if the review's RCTs are employing high or low intensity CBT as defined by Roth and Pilling's Department of Health report[3]. High intensity CBT refers to face to face therapy with a relatively specialist trained CBT therapist and low intensity is all other types of CBT (blended care, guided self-help, internet-based, structured exercises or brief interventions).

Comparator: We will include systematic reviews if they explore comparisons of CBT to either: 1) Active: a non-CBT comparator intervention 2) No Active: no intervention, waitlist control, placebo or treatment as usual or (3) Another format of CBT (e.g. computerised CBT versus face to face).

For peer review only - http://bmjopen.bmj.com/site/about/guidelines.xhtml

Outcomes: We will include systematic reviews which report information on at least one of the following patient or other reported outcomes: (1) HRQL (2) Psychological (3) Physical/physiological. We will include reviews with short (<12 months) and long term (>=12 months) outcomes.

Restrictions: We will include only reviews that are published/available in the English language due to the limited study timescale. We shall only include reviews which were published after 1992.

Information Sources

Our method of identifying systematic reviews will be conducted according to the principles of the Cochrane Handbook for Systematic Reviews of Interventions [14] and recommendations for conducting Overviews of Systematic Reviews [15].

The search strategy will be run across the Database of Abstracts of Reviews of Effects (DARE: up to March 2015), the Cochrane Library of Systematic Reviews, MEDLINE, EMBASE, PsycInfo, CINAHL, Child Development and Adolescent Studies (CDAS) and OpenGrey. This list was compiled by testing and searching the specificity and inclusivity of several databases and with the guidance of the ECG.

Search Strategy

A comprehensive search strategy comprising of free-text and controlled vocabulary terms identified by the ECG and from key papers from our preliminary scoping searches of systematic reviews on CBT will be run. We will use The Scottish Intercollegiate Guidelines Network (SIGN) systematic review filter available on the InterTASC Information Specialists' Sub-Group (ISSG) website[16], across Medline, Embase and CINAHL. We will use the McMaster's filter [17] within PsycInfo.

Our scoping work has identified that the earliest published review of CBT which has not been superseded is 1992[18]. This year also saw the advent of the Cochrane Collaboration, which implements high quality systematic reviews of RCTs across health care. Therefore, we restrict our search to the last 26 years.

Our search strategy picked up 36/36 sensitivity check papers. The strategy was adapted and checked for use across each of our selected databases. Our MEDLINE search strategy is attached in Appendix A.

We will perform an update search (April 2019) to check for any additional systematic reviews which have been published in the intervening year. We will also search PROSPERO, ClinicalTrials.gov and Clinical Trials Registry Platform (ICTRP) to identify any on-going systematic reviews and clinical trials to inform our discussion.

Study records

Data management

Search results will be exported into Endnote for de-duplication and then exported into Covidence, as recommended by Cochrane [19]. The full-text of reviews shortlisted for full text analysis will also be uploaded to Covidence. We shall perform data extraction using Microsoft Excel.

Selection process

Two reviewers will independently screen titles and abstracts using the abstract screening questionnaire which is based on the eligibility criteria. We will obtain full-text reports of those reviews selected for inclusion or for any uncertain cases. Two reviewers will independently perform review selection with the full text screening questionnaire, which includes the following reasons for exclusion (1) Not a systematic review, (2) Does not summarise RCT data separately (3) Does not report CBT specific data separately (4) CRD criteria (4 out of 5) not fulfilled (5) No HRQL, psychological or physical/physiological outcome (6) Full-text not available in English (7) Conference abstract with insufficient data. We will not contact authors for clarification. We will resolve any disagreements regarding the inclusion or exclusion of individual reviews by discussion with a third reviewer.

The search process and study identification will be documented in a figure as recommended by PRISMA statement[20]. This will result in a final list of included and excluded systematic reviews along with reasons for exclusion. This process will not be blinded so all reviewers will be able to see the authors and their affiliated institutions.

For peer review only

Data collection process

We developed a bespoke data extraction form with the ECG. Two reviewers will pilot the form on the first 18 reviews from the sensitivity check for the search strategy and revise accordingly. Two reviewers will extract the review data items and perform the AMSTAR-2 quality assessment. A third reviewer will compare the duplicate extractions and the anomalies will be discussed until a decision is reached.

Data items

The information extracted for each review will include study ICD-11 category of disease (primary or secondary level), aims, study design (systematic review of RCT or systematic review of RCT and non-RCT), risk of bias (Note whether the review used a risk of bias measure), number of RCTs and number of participants , demographics, intervention and control group description (category (high or low intensity), number of RCTs and number of session/frequency, duration), setting, and whether the review included HRQoL, depression, anxiety or a physical/physiological outcome (description). We shall make a free text list of all available outcomes reported in the review, in addition to those we specifically target. Descriptive information on mechanism data, acceptability, satisfaction, adverse events and economic analyses will also be extracted, when available.

We shall therefore emphasise the importance of long term (>=12 month follow-up) above short term (<12 month follow-up

Critical appraisal of included reviews

Each systematic review will be assessed independently by two reviewers using the AMSTAR 2[21] tool. We will not reassess the quality of the individual included RCTs. We will calculate the rate of agreement between the two reviewers and report. We will resolve any discrepancies with a third reviewer. Guidance suggests there are seven critical domains within the AMSTAR-2 items[21] and suggests categorising a review with 'high' confidence in the results of the review if we find no critical weakness and no or only one non-critical weakness; 'moderate' confidence if more than one non-critical weakness with no critical weakness; 'low' if there is one critical weakness with or without non-critical weaknesses and 'critically low' if there is more than one critical weakness with or without non-critical weaknesses[21].

Evidence Map

Overall map: We will produce a Bubble map[22] to represent the volume of systematic review data across all physical and mental health problems. The map will denote the total number of reviews (size of bubble), the total number of participants included in the reviews (y axis), the number of RCTs (x axis) by the primary physical or mental health (ICD-11 primary/secondary category) problem the review targets.

Mapping by health problem: Summary tables will present included review details grouped by ICD-11 categories. Information will include Intervention details, comparison group details, follow-up period, outcomes measured, effect size and confidence intervals for primary outcome / outcome pertaining to aim of review, number of RCTs, AMSTAR-2 rating, age and country. Within each health problem category we shall order reviews firstly by those which compared CBT to an active comparator and secondly those where it is compared to a non-active comparator.

Mapping by review details: The availability of the evidence will also be described by the following: (1) severity (mild, moderate, severe), (2) who (children, adults, older adults), (3) how (CBT intervention details), (4) when (prevention, standard treatment, relapse prevention etc), (5) where (primary, secondary, hospital setting), (6) psychological outcomes, (7) physiological outcomes and (8) HRQoL outcomes. The table aims to show the areas where systematic reviews have looked and where they have not. We propose to use the confidence ratings of AMSTAR-2[21] to code reviews with 'high confidence' (green), 'moderate confidence' (yellow), 'low confidence' (amber) and 'critically low' (red)[23]. This aims to give some direction as to the level of confidence.

Stage two

From the evidence maps populated in stage one we shall focus on the common outcomes examined within the included reviews. Stage two is to identify systematic reviews which we can synthesis to identify generic and specific effects of CBT across and within health problems.

For peer review only

Outcomes and prioritisation

Primary outcome

This overview will prioritise long term effects of CBT upon HRQL outcomes.

Secondary outcomes

Where no long term (>= 12 month) follow-up data is available we shall present the longest follow-up point available or the time point where the meta-analytic synthesis was performed. If there are separate analyses for several measurements of the same outcome then we will chose the analysis with the largest number of RCTs included. If they are equal then we will select the analysis of the measurement with the best psychometric properties.

We shall always extract data on HRQoL, depression, anxiety and one physical/physiological outcomes. If, in addition to or instead of HRQoL, depression and anxiety, there are multiple psychological and physical/physiological outcomes we will make a list of all available outcomes reported. If we find an additional common outcome, deemed meaningful by the ECG, which we have not focused on we can return to the review and extract this information.

If there are separate analyses for different classifications of response to treatment (response, recovery, relapse, remission) for the same outcome. We shall chose

- That which is identified as the primary outcome
- The analysis with the highest GRADE score (if available)
- The analysis which includes the greatest number of RCTs

Where available we will descriptively report the descriptions of mechanisms of action, patient satisfaction, adverse events and economic outcomes.

Selection process

We shall group all of the reviews which include a HRQoL outcome together. From these we shall identify those which have performed a meta-analysis of the data. These reviews shall be grouped by their ICD-11 categorisation (i.e. Neoplasms). At this stage we shall check if any of the included systematic reviews, within a health problem category, share primary RCTs. If we identify two or more reviews, which are eligible for inclusion but share the same primary RCTs we will use the following criteria hierarchy to choose one review for inclusion into the overview:

1. The review with the highest AMSTAR rating
2. The most recent review
3. The review with the larger number of studies included

We shall return to the full text of reviews that are selected and extract effect sizes, confidence intervals and heterogeneity measures. For effect sizes based on continuous outcome measures, the combined intervention/control group means, standard deviations and the total number of participants per group shall be extracted. For binary outcomes we shall extract from the combined intervention/control group the number of participants who have achieved the desired outcome plus the total number of participants.

The selected reviews will be examined to identify those with moderate clinical, design and statistical homogeneity. Statistical heterogeneity in treatment effect estimates between health problems will be explored using the I^2 statistic (moderate to low heterogeneity I^2 less than 75%); clinical heterogeneity will be explored through discussion with the ECG; and design heterogeneity explored using AMSTAR-2 scores.

We shall repeat this process for all reviews which include a depression outcome and an anxiety outcome. We will list all the physical/physiological outcomes which have been examined across all of our included reviews. The outcome which is the most common will be identified as the fourth outcome for selection.

Synthesis

We will synthesise these reviews and provide pooled treatment effects for all reviews which include a (1) HRQoL outcome, (2) Depression outcome (3) anxiety outcome and (4) most common physical/physiological outcome.

For peer review only - http://bmjopen.bmj.com/site/about/guidelines.xhtml

This formal quantitative data synthesis will be undertaken using a two-step frequentist approach to a PMA. This method provides a single pooled estimate of the treatment effect along with estimates of degree of heterogeneity between reviews. This allows for both between study variability within the health problem (if random effects meta-analysis was used in the original indication review) and between health problem variability (using random effects), but does assume exchangeability of treatment effects.

We will perform this process for the outcomes of HRQoL, depression, anxiety and the most common physical/ physiological outcome. As we have collected other psychological and physical/physiological outcomes we will remain flexible and will consider additional synthesis suggested by the ECG.

Sub-group analysis:

For each of our key outcomes (HRQoL, depression, anxiety and the most common physical outcome) we will perform a sub-group analysis comparing (1) reviews which include RCTs with high intensity CBT (2) those with low intensity CBT, (3) those with a mixture of high and low intensity CBT RCTs. In addition, if we find reviews which directly compare high and low intensity CBT within the review we shall group these and if possible pool the results; comparing high to low intensity CBT groups rather than intervention to control groups.

We do not plan to perform any further sub-group analyses however if the data is suitable we are flexible to additional analyses (e.g. by control group type or follow-up period) if the comparison is deemed important by the ECG once we have reviewed the available data.

Publication bias

This will be assessed per outcome therefore if we have more than 10 systematic reviews per outcome (HRQoL, Depression, Anxiety and the most common physical outcome) then the evidence of funnel plot asymmetry will be assessed using both the funnel plot and the Egger test using a conservative P-value of 0.1 to acknowledge the low power of this test.

Summary

We are sensitive to the importance of not overstating conclusions representing CBT as being effective or not and to accurately reflect where further research, whether primary or secondary analysis work is needed. We will caveat all summary statements and recommendations with the limitations of the methodology but treat this as a necessary step in addressing the current state of the CBT evidence base.

The mapping exercise will identify in which health problems, across which sub-groups, contexts and with what format, CBT has been evaluated, thereby identifying gaps which have not been examined with a high quality systematic review.

The synthesis stage can identify if CBT can produce long term changes in quality of life. It will also present, with varying degrees of confidence, where CBT does or does not produce generic or problem-specific long term changes upon specific functions.

We will search Prospero, ClinicalTrials.gov and ICTRP to identify on-going trials or systematic reviews which have addressed the areas we recommend for further research. This summary will lead to a set of recommendations regarding the prioritisation of primary or secondary research into areas where we cannot generalise the clinical effectiveness findings and the evidence base is weak.

Dissemination plan

An overview of the project will be published in the NIHR journals library. We plan to prepare secondary publications detailing the generic effects of CBT upon HRQL, depression, anxiety and the most commonly found physical/physiological outcome. When there is sufficient data we will publish health problem specific overview papers. We hope to present the findings at international conferences to make sure the information is communicated to the patient population perhaps via patient conferences and/or social media.

For peer review only

References

1. Grazebrook KaG, A. What are cognitive and/or behavioural psychotherapies. UKCP/BACP Mapping psychotherapy exercise. . 2005.
2. Mansell W. The seven C's of CBT: A consideration of the future challenges for cognitive behaviour therapy. *Behavioural and Cognitive Psychotherapy.* 2008;.36(6):pp.
3. Roth A, Pilling S. *The competencies required to deliver effective Cognitive and Behavioural Therapy for people with Depression and Anxiety disorders. .* London: Department of Health.; 2007.
4. Lockwood C, Page T, Conroy-Hiller T. Effectiveness of individual therapy and group therapy in the treatment of schizophrenia. *International Journal of Evidence-Based Healthcare.* 2004;.2(10):pp.
5. Richmond H, Hall AM, Copsey B, et al. The Effectiveness of Cognitive Behavioural Treatment for Non-Specific Low Back Pain: A Systematic Review and Meta-Analysis. *Plos One.* 2015;10(8).
6. Ballesio A, Aquino MRJV, Feige B, et al. The effectiveness of behavioural and cognitive behavioural therapies for insomnia on depressive and fatigue symptoms: A systematic review and network meta-analysis. *Sleep Medicine Reviews.* 2018:114-129.
7. Brasure M, Fuchs E, MacDonald R, et al. Psychological and Behavioral Interventions for Managing Insomnia Disorder: An Evidence Report for a Clinical Practice Guideline by the American College of Physicians. *Ann Intern Med.* 2016;165(2):113-124.
8. Johnson JA, Rash JA, Campbell TS, et al. A systematic review and meta-analysis of randomized controlled trials of cognitive behavior therapy for insomnia (CBT-I) in cancer survivors. *Sleep Med Rev.* 2016;27:20-28.
9. Burns AM, Erickson DH, Brenner CA. Cognitive-behavioral therapy for medication-resistant psychosis: a meta-analytic review. *Psychiatr Serv.* 2014;65(7):874-880.
10. Cormac I, Jones C, Campbell C. Cognitive behaviour therapy for schizophrenia. *The Cochrane database of systematic reviews.* 2002(1):CD000524.
11. Durham RC CJ, Power KG, Sharp DM, Macdonald RR, Major KA, et al. Long-term outcome of cognitive behaviour therapy clinical trials in central Scotland.

. *Health Technol Assess.*9(42).
12. Perlis RH. Cognitive behavioural therapy has short term but not long term benefits in people with residual symptoms of depression. *Evidence-based mental health.* 2005;8(3):75.
13. CRD. *Undertaking Systematic Reviews of Research on Effectiveness: CRD's Guidance for Carrying Out or Commissioning Reviews (CRD Report 4) 2nd edn. .* York: NHS Centre for Reviews and Dissemination, University of York.; 2001.
14. Higgins JP, Green S. *Cochrane handbook for systematic reviews of interventions.* Vol 5: Wiley Online Library; 2008.
15. Cochrane. The Cochrane Comparing Multiple Interventions Group

Accessed 6th Jan 2016.
16. ISSG. Filters to Identify Systematic Reviews. https://sites.google.com/a/york.ac.uk/issg-search-filters-resource/filters-to-identify-systematic-reviews. Accessed 3 August, 2016.
17. University M. Search Strategies for PsycINFO in Ovid Syntax 2016; https://hiru.mcmaster.ca/hiru/HIRU_Hedges_PsycINFO_Strategies.aspx. Accessed 10 January 2018.
18. Grossman PBaH, J N. Self-control interventions with internalizing disorders: A review and analysis. *School Psychology Review.* 1992;21(2):229-245.
19. Covidence. Covidence: Faster systematic reviews. https://www.covidence.org/. Accessed June, 2016.
20. Moher D, Liberati A, Tetzlaff J, Altman DG, Grp P. Preferred Reporting Items for Systematic Reviews and Meta-Analyses: The PRISMA Statement. *Plos Med.* 2009;6(7).

For peer review only

21. Shea BJ, Reeves BC, Wells G, et al. AMSTAR 2: a critical appraisal tool for systematic reviews that include randomised or non-randomised studies of healthcare interventions, or both. *BMJ.* 2017;358:j4008.
22. Hempel S, Shekelle PG, Taylor SL, et al. Evidence map of mindfulness. *VA-ESP Project #05-226.* 2014.
23. Shea R, Wells, Thuku, Hamel. AMSTAR 2: a critical appraisal tool for systematic reviews that include randomised or non-randomised studies of healthcare interventions, or both. *BMJ.* 2017.
24. Howick J, Glasziou P, Aronson JK. Evidence-based mechanistic reasoning. *J Roy Soc Med.* 2010;103(11):433-441.

For peer review only

Appendix A: Search Strategy for MEDLINE

Database & platform: Ovid MEDLINE(R) Epub Ahead of Print, In-Process & Other Non-Indexed Citations, Ovid MEDLINE(R) Daily and Ovid MEDLINE(R) 1946 to Present

Search strategy development date: 9 March 2018

1. (cognitive adj2 behavio?r adj3 (therap$ or theor$ or intervention$ or train$ or treatment$ or psychotherap$ or programme$ or program$ or method$ or approach$)).ti,ab,kw.

2. (cognitive adj2 behavio?ral adj3 (therap$ or theor$ or intervention$ or train$ or treatment$ or psychotherap$ or programme$ or program$ or method$ or approach$)).ti,ab,kw.

3. CBT.ti,ab,kw.

4. Cognitive Therapy/

5. or/1-4

6. Meta-Analysis as Topic/

7. meta analy$.tw.

8. metaanaly$.tw.

9. Meta-Analysis/

10. (systematic adj (review$1 or overview$1)).tw.

11. exp Review Literature as Topic/

12 or/6-11

13. cochrane.ab.

14. embase.ab.

15. (psychlit or psyclit).ab.

16. (psychinfo or psycinfo).ab.

17. (cinahl or cinhal).ab.

18. science citation index.ab.

19. bids.ab.

20. cancerlit.ab.

21. or/13-20

22. reference list$.ab.

23. bibliograph$.ab.

24. hand-search$.ab.

25. relevant journals.ab.

26. manual search$.ab.

27. or/22-26

28. selection criteria.ab.

For peer review only

29. data extraction.ab.

30. 28 or 29

31. Review/

32. 30 and 31

33. Comment/

34. Letter/

35. Editorial/

36. animal/

37. human/

38. 36 and 37

39. 36 not 38

40. or/33-35,39

41. 12 or 21 or 27 or 32

42. 41 not 40

43. 5 and 42

44. limit 43 to yr="1992-2018"

For peer review only

PRISMA-P 2015 Checklist

This checklist has been adapted for use with protocol submissions to *Systematic Reviews* from Table 3 in Moher D et al: Preferred reporting items for systematic review and meta-analysis protocols (PRISMA-P) 2015 statement. *Systematic Reviews* 2015 **4**:1

Section/topic	#	Checklist item	Information reported		Line number(s)
			Yes	No	
**ADMINISTRATIVE INFORMATION**					
**Title**					
Identification	1a	Identify the report as a protocol of a systematic review	☒	☐	1
Update	1b	If the protocol is for an update of a previous systematic review, identify as such	☐	☐	Not applicable
**Registration**	2	If registered, provide the name of the registry (e.g., PROSPERO) and registration number in the Abstract	☒	☐	64-66
**Authors**					
Contact	3a	Provide name, institutional affiliation, and e-mail address of all protocol authors; provide physical mailing address of corresponding author	☒	☐	4-21
Contributions	3b	Describe contributions of protocol authors and identify the guarantor of the review	☒	☐	23-27
**Amendments**	4	If the protocol represents an amendment of a previously completed or published protocol, identify as such and list changes; otherwise, state plan for documenting important protocol amendments	☐	☐	Not applicable
**Support**					
Sources	5a	Indicate sources of financial or other support for the review	☒	☐	37-41
Sponsor	5b	Provide name for the review funder and/or sponsor	☒	☐	29-35
Role of sponsor/funder	5c	Describe roles of funder(s), sponsor(s), and/or institution(s), if any, in developing the protocol	☒	☐	34-35
**INTRODUCTION**					
**Rationale**	6	Describe the rationale for the review in the context of what is already known	☒	☐	112-118
**Objectives**	7	Provide an explicit statement of the question(s) the review will address with reference to	☒	☐	107-110 & 119-128

BioMed Central
The Open Access Publisher

For peer review only

For peer review only - http://bmjopen.bmj.com/site/about/guidelines.xhtml

Section/topic	#	Checklist item	Information reported		Line number(s)
			Yes	No	
		participants, interventions, comparators, and outcomes (PICO)			
**METHODS**					
**Eligibility criteria**	8	Specify the study characteristics (e.g., PICO, study design, setting, time frame) and report characteristics (e.g., years considered, language, publication status) to be used as criteria for eligibility for the review	☒	☐	145-181
**Information sources**	9	Describe all intended information sources (e.g., electronic databases, contact with study authors, trial registers, or other grey literature sources) with planned dates of coverage	☒	☐	182-206
**Search strategy**	10	Present draft of search strategy to be used for at least one electronic database, including planned limits, such that it could be repeated	☒	☐	Appendix A
STUDY RECORDS					
Data management	11a	Describe the mechanism(s) that will be used to manage records and data throughout the review	☒	☐	207-211
Selection process	11b	State the process that will be used for selecting studies (e.g., two independent reviewers) through each phase of the review (i.e., screening, eligibility, and inclusion in meta-analysis)	☒	☐	212-225
Data collection process	11c	Describe planned method of extracting data from reports (e.g., piloting forms, done independently, in duplicate), any processes for obtaining and confirming data from investigators	☒	☐	226-230
**Data items**	12	List and define all variables for which data will be sought (e.g., PICO items, funding sources), any pre-planned data assumptions and simplifications	☒	☐	231-242
**Outcomes and prioritization**	13	List and define all outcomes for which data will be sought, including prioritization of main and additional outcomes, with rationale	☒	☐	278-299
**Risk of bias in individual studies**	14	Describe anticipated methods for assessing risk of bias of individual studies, including whether this will be done at the outcome or study level, or both; state how this information will be used in data synthesis	☒	☐	243-251
DATA					
**Synthesis**	15a	Describe criteria under which study data will be quantitatively synthesized	☒	☐	325-337
	15b	If data are appropriate for quantitative synthesis, describe planned summary measures, methods of handling data, and methods of combining data from studies, including any planned exploration of consistency (e.g., I^2, Kendall's tau)	☒	☐	300-323
	15c	Describe any proposed additional analyses (e.g., sensitivity or subgroup analyses, meta-	☒	☐	339-348

BioMed Central The Open Access Publisher

For peer review only - http://bmjopen.bmj.com/site/about/guidelines.xhtml

Section/topic	#	Checklist item	Information reported		Line number(s)
			Yes	No	
		regression)			
	15d	If quantitative synthesis is not appropriate, describe the type of summary planned	☒	☐	253-271
**Meta-bias(es)**	16	Specify any planned assessment of meta-bias(es) (e.g., publication bias across studies, selective reporting within studies)	☒	☐	349-353
**Confidence in cumulative evidence**	17	Describe how the strength of the body of evidence will be assessed (e.g., GRADE)	☒	☐	316-323

For peer review only

BioMed Central
The Open Access Publisher

BMJ Open

Effectiveness of Cognitive Behavioural Therapy: A protocol for an overview of systematic reviews and meta-analyses

Journal:	*BMJ Open*
Manuscript ID	bmjopen-2018-025761.R1
Article Type:	Protocol
Date Submitted by the Author:	08-Oct-2018
Complete List of Authors:	Fordham, Beth; University of Oxford Nuffield Department of Orthopaedics Rheumatology and Musculoskeletal Sciences<br>Sugavanam, Thavapriya; University of Oxford Nuffield Department of Orthopaedics Rheumatology and Musculoskeletal Sciences,<br>Hopewell, Sally; University of Oxford, Centre for Statistics in Medicine, Nuffield Department of Orthopaedics, Rheumatology and Musculoskeletal Sciences<br>Hemming, Karla; University of Birmingham, Public Health<br>Howick, Jeremy; University of Oxford, Primary Care Health Sciences<br>Kirtley, Shona; University of Oxford, Centre for Statistics in Medicine<br>dasNair, Roshan; Institute of Mental Health, University of Nottingham<br>Hamer-Hunt, Julia<br>Lamb, Sarah; University of Oxford Nuffield Department of Orthopaedics Rheumatology and Musculoskeletal Sciences
**Primary Subject Heading**:	Evidence based practice
Secondary Subject Heading:	Mental health, Health services research
Keywords:	Cognitive behaviour therapy, Overview of systematic reviews, MENTAL HEALTH

SCHOLARONE™ Manuscripts

Effectiveness of Cognitive Behavioural Therapy: A protocol for an overview of systematic reviews and meta-analyses

Beth Fordham [1], Thavapriya Sugavanam [1], Sally Hopewell [2], Karla Hemming [3], Jeremy Howick [4], Shona Kirtley [2], Roshan das Nair [5], Julia Hamer-Hunt [6], and Sarah E Lamb [1]

Corresponding author: Beth Fordham beth.fordham@ndorms.ox.ac.uk [1] Nuffield Department of Orthopaedics, Rheumatology and Musculoskeletal Sciences, University of Oxford, Botnar Research Centre, Windmill Road, Headington, Oxford OX3 7LD

Author affiliations: [2] Centre for Statistics in Medicine, Nuffield Department of Orthopaedics, Rheumatology and Musculoskeletal Sciences, University of Oxford. [3]Public Health, Epidemiology and Biostatistics, Institute of Applied Health Research, University of Birmingham. [4]Department of Primary Care Health Sciences, Centre for Evidence-Based Medicine, University of Oxford, [5]Institute of Mental Health, University of Nottingham, [6]Patient representative.

Email: Thavapriya Sugavanam priya.sugavanam@ndorms.ox.ac.uk, Sally Hopewell sally.hopewell@csm.ox.ac.uk, Karla Hemming K.Hemming@bham.ac.uk, Jeremy Howick jeremy.howick@phc.ox.ac.uk, Shona Kirtley shona.kirtley@csm.ox.ac.uk, Roshan das Nair Roshan.Dasnair@nottingham.ac.uk, Julia Hamer-Hunt julia.hamerhunt@gmail.com and Sallie Lamb sarah.lamb@ndorms.ox.ac.uk.

Authors' contributions: BF is the guarantor. TS developed the protocol with BF. SH provided expertise on design and methodology. KH provided expertise on methodology in particular panoramic meta-analysis. JH provided expertise in methodology. SK developed the draft search strategy plan. RdN, and JHH provided clinical contextual feedback. SL provided expertise in all areas of design, methodology and rigour. All authors read, provided feedback and approved the final manuscript.

Funding statement: This overview of systematic reviews is funded by the National Institute for Health Research, Health Technology Assessment Programme (funding reference number HTA 15/174: Cognitive Behavioural Therapy: An overview of systematic reviews and meta-analyses). The University of Oxford is the sponsor of this overview of systematic reviews. The NIHR HTA programme is funding this overview of systematic reviews. They will provide funding to NDORMS (University of Oxford) who will carry out the research and store the data. The NIHR HTA programme is not involved in any other aspect of the project design, data collection or analyses. They will be made aware of the findings at milestones throughout the project.

Acknowledgments:
Professor Sallie Lamb receives funding from the National Institute for Health Research (NIHR) Collaboration for Leadership in Applied Health Research and Care Oxford at Oxford Health NHS Foundation Trust. The views expressed are those of the author(s) and not necessarily those of the NHS, the NIHR or the Department of Health and Social Care.

Competing Interests statement:
No authors have any competing interests to declare.

Word count: 3557words

For peer review only

Abstract

Introduction: Cognitive behavioural therapy (CBT) is a psychological therapy which has been used to improve patient well-being across multiple mental and physical health problems. Its effectiveness has been examined in thousands of randomised control trials which have been synthesised into hundreds systematic reviews. The aim of this overview is to map, synthesise and assess the reliability of evidence generated from these systematic reviews of the effectiveness of CBT across all health conditions, patient groups and settings.

Methods and analysis: We will run our search strategy, to identify systematic reviews of CBT, within Database of Abstracts of Reviews of Effects, the Cochrane Library of Systematic Reviews, MEDLINE, EMBASE, PsycInfo, CINAHL, Child Development and Adolescent Studies and OpenGrey between January 1992 and 25th April 2018. Independent reviewers will sift, perform data extraction in duplicate and assess the quality of the reviews using the Assessing the Methodological Quality of Systematic Reviews (version.2) tool. The outcomes of interest include: health related quality of life, depression, anxiety, psychosis and physical/physiological outcomes prioritised in the individual reviews. The evidence will be mapped and synthesized where appropriate by health problem, patient sub-groups, intervention type, context and outcome.

Ethics and dissemination: Ethical approval is not required as this is an overview of published systematic reviews. We plan to publish results in peer reviewed journals, present at international and national academic, clinical and patient conferences.

Registration details:
Our protocol was registered with the International Prospective Register of Systematic Reviews (PROSPERO) on 17th April 2018 (registration number: CRD42017078690).

Strengths and limitations of this study

- A strength of this study is that it is the only up to date overview of systematic reviews examining randomised control trials of the effectiveness of Cognitive Behavioural Therapy across all health problems, populations and settings.
- Another strength is that our method allows us to map the available evidence and develop a framework to suggest where evidence can be generalised to and from.
- The main weakness is that we will only include systematic reviews which explicitly state, “Cognitive behavioural therapy” (including all synonyms) in their abstract, title or keywords. This excludes broader reviews which encapsulate the CBT within “psychological interventions.”
- Another weakness is that, we are reliant on the information provided in the systematic reviews therefore we might omit RCTs if they are not included in the reviews we synthesis.

For peer review only

Introduction

The cognitive behavioural model theorises that the way in which we think and behave will influence our emotional and physical well-being and consequently our overall quality of life. The relationships between cognitions, behaviours, emotions and physical responses are all considered bidirectional[1,2].

Cognitive Behavioural Therapy (CBT) is a talking therapy, which targets identifying maladaptive thoughts and behaviours and challenging them, trying to develop different ways of thinking and acting to improve the emotional and physical outcomes for patients. CBT has a core set of competencies which can be applied trans-diagnostically, however it has also been tailored for use in specific populations, such as CBT-Insomnia. Most CBT is delivered in adherence with CBT process manuals specific to the health problem. Roth and Pilling, on behalf of the Department of Health, developed a set of core competencies for CBT and included a division between high and low intensity CBT [3]. They defined high intensity as formal CBT with a CBT-trained health professional predominantly delivered face to face in an individual or group format. Low intensity interventions focus on patient self-help and can be delivered by health professionals with very little to fairly comprehensive CBT training and via several platforms (internet, phone, paper-based). This distinction can become less clear in some forms of CBT, called "blended care", where high intensity therapy is combined with low intensity self-help methods.

The effectiveness of CBT has been evaluated with randomised control trials (RCTs) , which have been synthesised into systematic reviews across numerous physical and mental health problems from schizophrenia[4] to low back pain[5]. We recognised some consistency across the CBT systematic reviews e.g. improving symptoms of insomnia in adults with various health problems [6], [7], [8]. However, we also identified areas with conflicting evidence for example with regards to the efficacy of CBT in reducing relapse in schizophrenia [9,10].

Whilst we are cognisant the volume and variety of available systematic reviews of CBT we are not aware of the quality of the reviews conducted across different health problems, populations and settings. Another limitation of current evidence, is that short term changes to function as a result of CBT do not guarantee long term changes[11,12] and much of the evidence focuses on shorter term outcomes.

This overview will explore the effects of CBT across all health problems, in all populations and in all settings. The primary outcome will be Health Related Quality of Life (HRQL) with the aim of capturing, to some degree, the broader, general, biopsychosocial influence of CBT in addition to its impact upon the specific functional outcomes.

Rationale

Our scoping work suggests there are more than 500 systematic reviews of CBT and there has been no published overview of systematic reviews since 2004. We will address this gap, and aim to map for which populations there are systematic reviews of RCTs examining CBT and document how well these reviews were conducted. Within each population we will identify whether (a) there is a need for new or better quality systematic reviews or RCTs or (b) that CBT worsens/does not alter/improves generic (HRQL) and problem specific health outcomes in comparison to active or not active control conditions in the short or long term follow-up period.

Objectives

The specific objectives include:

(1) Stage one: A Map of the evidence
   a. Map and assess the quality of available evidence
(2) Stage two: A synthesis of the evidence
   a. A descriptive and a panoramic meta-analytic synthesis of the evidence (PMA) by ICD-11 health problem categorisations and by common outcomes (HRQL, depression, anxiety, psychosis and the most common physical/physiological outcome).
   b. Sub-group analysis to explore high versus low intensity CBT (as defined by Roth and Pilling, 2007[3]) for a health problem.

For peer review only - http://bmjopen.bmj.com/site/about/guidelines.xhtml

Methods

Patient and Public Involvement

We are working with a CBT expert consultation group (ECG) consisting of clinical academics (n=7), research academics (n=9) and service users (n=4). We meet with this group face to face twice and communicate via phone/email throughout the overview process to guide our protocol development, synthesis strategy, and interpretation. We hope the ECG will guide our overview to produce clinically meaningful outputs. The group will not be involved in any of the data extraction or quality assessment to ensure no undue influence.

Methods

We shall perform two stages within this overview. Stage one is to identify all the available systematic reviews of CBT, which include RCT evidence then to map the available evidence along with a quality assessment of the included reviews. The second stage will be to meaningfully synthesise the evidence by common outcomes across health problems and to specifically examine the comparative effectiveness of high and low intensity CBT.

Stage one: Mapping the evidence

This stage will detail how we will identify and select the systematic reviews for inclusion in order to generate a comprehensive map of the evidence.

Eligibility criteria

To be included in the evidence map and overview of systematic reviews, studies must meet the following criteria:

Type of studies: We will include systematic reviews of randomised control trials (RCTs) which evaluate the effects of CBT. We will include systematic reviews which include both randomised and non-randomised trials so long as the review has summarised the RCT evidence independently.

To be included, systematic reviews must fulfil a minimum of 4 methodological criteria as defined by the Centre for Reviews and Dissemination (CRD), University of York, as part of the Database of Reviews of Effects (DARE) database (http://www.crd.york.ac.uk/crdweb) [13]: (1) inclusion/exclusion criteria reported; (2) adequate search strategy; (3) included studies synthesised; (4) quality of the included studies assessed; (5) sufficient details about the included studies reported. The University of York have provided us with detailed definitions for each of these criteria. For example the minimum sufficient details of the individual studies would be details of the population, setting, interventions and results for every included study (in text, tables or online appendices).

Type of participants: We will include systematic reviews of RCTs, which include data from all age groups and any gender. We will include all health problems recognised within the ICD-11.

Setting: We will include systematic reviews of RCTs that have been conducted in any context or country.

Intervention: We will only include systematic reviews where Cognitive Behavioural Therapy (or other CBT synonyms) has been explicitly reported in the review title, abstract or key words. We will include all formats of CBT. We will classify if the review's RCTs are employing high or low intensity CBT as defined by Roth and Pilling's Department of Health report[3]. High intensity CBT refers to face to face therapy with a relatively specialist trained CBT therapist and low intensity is all other types of CBT (blended care, guided self-help, internet-based, structured exercises or brief interventions).

Comparator: We will include systematic reviews if they explore comparisons of CBT to either: 1) Active: a non-CBT comparator intervention 2) No Active: no intervention, waitlist control, placebo or treatment as usual or (3) Another format of CBT (e.g. computerised CBT versus face to face).

Outcomes: We will include systematic reviews which report information on at least one of the following patient or other reported outcomes: (1) HRQL (2) Psychological (3) Physical/physiological. We will include reviews with short (<12 months) and long term (>=12 months) outcomes.

For peer review only

Restrictions: We will include only reviews that are published/available in the English language due to the limited study timescale. We shall only include reviews which were published after 1992.

Information Sources

Our method of identifying systematic reviews will be conducted according to the principles of the Cochrane Handbook for Systematic Reviews of Interventions [14] and recommendations for conducting Overviews of Systematic Reviews [15].

The search strategy will be run across the Database of Abstracts of Reviews of Effects (DARE: up to March 2015), the Cochrane Library of Systematic Reviews, MEDLINE, EMBASE, PsycInfo, CINAHL, Child Development and Adolescent Studies (CDAS) and OpenGrey. This list was compiled by testing and searching the specificity and inclusivity of several databases and with the guidance of the ECG.

Search Strategy

A comprehensive search strategy comprising of free-text and controlled vocabulary terms identified by the ECG and from key papers from our preliminary scoping searches of systematic reviews on CBT will be run. We will use The Scottish Intercollegiate Guidelines Network (SIGN) systematic review filter available on the InterTASC Information Specialists' Sub-Group (ISSG) website[16], across Medline, Embase and CINAHL. We will use the McMaster's filter [17] within PsycInfo.

Our scoping work has identified that the earliest published review of CBT which has not been superseded is 1992[18]. This year also saw the advent of the Cochrane Collaboration, which implements high quality systematic reviews of RCTs across health care. Therefore, we restrict our search to the last 26 years.

Our search strategy picked up 36/36 sensitivity check papers. The strategy was adapted and checked for use across each of our selected databases. Our MEDLINE search strategy is attached in Appendix A.

We will perform an update search (April 2019) to check for any additional systematic reviews which have been published in the intervening year. We will also search PROSPERO, ClinicalTrials.gov and Clinical Trials Registry Platform (ICTRP) to identify any on-going systematic reviews and clinical trials to inform our discussion.

Study records

Data management

Search results will be exported into Endnote for de-duplication and then exported into Covidence, as recommended by Cochrane [19]. The full-text of reviews shortlisted for full text analysis will also be uploaded to Covidence. We shall perform data extraction using Microsoft Excel.

Selection process

Two reviewers will independently screen titles and abstracts using the abstract screening questionnaire which is based on the eligibility criteria. We will obtain full-text reports of those reviews selected for inclusion or for any uncertain cases. Two reviewers will independently perform review selection with the full text screening questionnaire, which includes the following reasons for exclusion (1) Not a systematic review, (2) Does not summarise RCT data separately (3) Does not report CBT specific data separately (4) CRD criteria (4 out of 5) not fulfilled (5) No HRQL, psychological or physical/physiological outcome (6) Full-text not available in English (7) Conference abstract with insufficient data. We will not contact authors for clarification. We will resolve any disagreements regarding the inclusion or exclusion of individual reviews by discussion with a third reviewer.

The search process and study identification will be documented in a figure as recommended by PRISMA statement[20]. This will result in a final list of included and excluded systematic reviews along with reasons for exclusion. This process will not be blinded so all reviewers will be able to see the authors and their affiliated institutions.

Data collection process

We developed a bespoke data extraction form with the ECG. Two reviewers will pilot the form on the first 18 reviews from the sensitivity check for the search strategy and revise accordingly. Two reviewers will extract the review data items and perform the AMSTAR-2 quality assessment. A third reviewer will compare the duplicate extractions and the anomalies will be discussed until a decision is reached.

For peer review only

Data items

The information extracted for each review will include study ICD-11 category of disease (primary or secondary level), aims, study design (systematic review of RCT or systematic review of RCT and non-RCT), risk of bias (Note whether the review used a risk of bias measure), number of RCTs and number of participants , demographics, intervention and control group description (category (high or low intensity), number of RCTs and number of session/frequency, duration), setting, and whether the review included HRQoL, depression, anxiety, psychosis or a physical/physiological outcome (description). We shall make a free text list of all available outcomes reported in the review, in addition to those we specifically target. Descriptive information on mechanism data, acceptability, satisfaction, adverse events and economic analyses will also be extracted, when available.

We shall therefore emphasise the importance of long term (>=12 month follow-up) above short term (<12 month follow-up

Critical appraisal of included reviews

Each systematic review will be assessed independently by two reviewers using the AMSTAR 2[21] tool. We will not reassess the quality of the individual included RCTs. We will calculate the rate of agreement between the two reviewers and report. We will resolve any discrepancies with a third reviewer. Guidance suggests there are seven critical domains within the AMSTAR-2 items[21] and suggests categorising a review with 'high' confidence in the results of the review if we find no critical weakness and no or only one non-critical weakness; 'moderate' confidence if more than one non-critical weakness with no critical weakness; 'low' if there is one critical weakness with or without non-critical weaknesses and 'critically low' if there is more than one critical weakness with or without non-critical weaknesses[21].

Evidence Map

<u>Overall map</u>: We will produce a Bubble map[22] to represent the volume of systematic review data across all physical and mental health problems. The map will denote the total number of reviews (size of bubble), the total number of participants included in the reviews (y axis), the number of RCTs (x axis) by the primary physical or mental health (ICD-11 primary/secondary category) problem the review targets.

<u>Mapping by health problem</u>: Summary tables will present included review details grouped by ICD-11 categories. Information will include Intervention details, comparison group details, follow-up period, outcomes measured, effect size and confidence intervals for primary outcome / outcome pertaining to aim of review, number of RCTs, AMSTAR-2 rating, age and country. Within each health problem category we shall order reviews firstly by those which compared CBT to an active comparator and secondly those where it is compared to a non-active comparator.

<u>Mapping by review details</u>: The availability of the evidence will also be described by the following: (1) severity (mild, moderate, severe), (2) who (children, adults, older adults), (3) how (CBT intervention details), (4) when (prevention, standard treatment, relapse prevention etc), (5) where (primary, secondary, hospital setting), (6) psychological outcomes, (7) physiological outcomes and (8) HRQoL outcomes. The table aims to show the areas where systematic reviews have looked and where they have not. We shall highlight any Individual Patient Data (IDP) meta-analyses. We propose to use the confidence ratings of AMSTAR-2[21] to code reviews with 'high confidence' (green), 'moderate confidence' (yellow), 'low confidence' (amber) and 'critically low' (red)[23]. This aims to give some direction as to the level of confidence.

<u>Stage two</u>

From the evidence maps populated in stage one we shall focus on the common outcomes examined within the included reviews. Stage two is to identify systematic reviews which we can synthesis to identify generic and specific effects of CBT across and within health problems.

Outcomes and prioritisation

<u>Primary outcome</u>

This overview will prioritise long term effects of CBT upon HRQL outcomes.

<u>Secondary outcomes</u>

For peer review only - http://bmjopen.bmj.com/site/about/guidelines.xhtml

Where no long term (>= 12 month) follow-up data is available we shall present the longest follow-up point available or the time point where the meta-analytic synthesis was performed. If there are separate analyses for several measurements of the same outcome then we will chose the analysis with the largest number of RCTs included. If they are equal then we will select the analysis of the measurement with the best psychometric properties.

We shall always extract data on HRQoL, depression, anxiety, psychosis and one physical/physiological outcomes. If, in addition to or instead of HRQoL, depression, anxiety and psychosis there are multiple psychological and physical/physiological outcomes we will make a list of all available outcomes reported. If we find an additional common outcome, deemed meaningful by the ECG, which we have not focused on we can return to the review and extract this information.

If there are separate analyses for different classifications of response to treatment (response, recovery, relapse, remission) for the same outcome. We shall chose

- That which is identified as the primary outcome
- The analysis with the highest GRADE score (if available)
- The analysis which includes the greatest number of RCTs

Where available we will descriptively report the descriptions of mechanisms of action, patient satisfaction, adverse events and economic outcomes.

Selection process

We shall group all of the reviews which include a HRQoL outcome together. From these we shall identify those which have performed a meta-analysis of the data. These reviews shall be grouped by their ICD-11 categorisation (i.e. Neoplasms). At this stage we shall check if any of the included systematic reviews, within a health problem category, share primary RCTs. If we identify two or more reviews, which are eligible for inclusion but share the same primary RCTs we will use the following criteria hierarchy to choose one review for inclusion into the overview:

1. The review with the highest AMSTAR rating
2. The most recent review
3. The review with the larger number of studies included

We shall return to the full text of reviews that are selected and extract effect sizes, confidence intervals and heterogeneity measures. For effect sizes based on continuous outcome measures, the combined intervention/control group means, standard deviations and the total number of participants per group shall be extracted. For binary outcomes we shall extract from the combined intervention/control group the number of participants who have achieved the desired outcome plus the total number of participants.

The selected reviews will be examined to identify those with moderate clinical, design and statistical homogeneity. Statistical heterogeneity in treatment effect estimates between health problems will be explored using the I^2 statistic (moderate to low heterogeneity I^2 less than 75%); clinical heterogeneity will be explored through discussion with the ECG; and design heterogeneity explored using AMSTAR-2 scores.

We shall repeat this process for all reviews which include a depression outcome, an anxiety outcome and a psychosis outcome. We will list all the physical/physiological outcomes which have been examined across all of our included reviews. The outcome which is the most common will be identified as the fifth outcome for selection.

Synthesis

We will synthesise these reviews and provide pooled treatment effects for all reviews which include a (1) HRQoL outcome, (2) Depression outcome (3) anxiety outcome (4) psychosis outcome and (5) most common physical/physiological outcome.

This formal quantitative data synthesis will be undertaken using a two-step frequentist approach to a PMA. This method provides a single pooled estimate of the treatment effect along with estimates of degree of heterogeneity between reviews. This allows for both between study variability within the health problem (if

For peer review only - http://bmjopen.bmj.com/site/about/guidelines.xhtml

random effects meta-analysis was used in the original indication review) and between health problem variability (using random effects), but does assume exchangeability of treatment effects.

We will perform this process for the outcomes of HRQoL, depression, anxiety, psychosis and the most common physical/ physiological outcome. As we have collected other psychological and physical/physiological outcomes we will remain flexible and will consider additional synthesis suggested by the ECG.

Sub-group analysis:

For each of our key outcomes (HRQoL, depression, anxiety, psychosis and the most common physical outcome) we will perform a sub-group analysis comparing (1) reviews which include RCTs with high intensity CBT (2) those with low intensity CBT, (3) those with a mixture of high and low intensity CBT RCTs. In addition, if we find reviews which directly compare high and low intensity CBT within the review we shall group these and if possible pool the results; comparing high to low intensity CBT groups rather than intervention to control groups.

We do not plan to perform any further sub-group analyses however if the data is suitable we are flexible to additional analyses (e.g. by control group type or follow-up period) if the comparison is deemed important by the ECG once we have reviewed the available data.

Publication bias

This will be assessed per outcome therefore if we have more than 10 systematic reviews per outcome (HRQoL, Depression, Anxiety, Psychosis and the most common physical outcome) then the evidence of funnel plot asymmetry will be assessed using both the funnel plot and the Egger test using a P-value of 0.1 to acknowledge the low power of this test.

Summary

We are sensitive to the importance of not overstating conclusions representing CBT as being effective or not and to accurately reflect where further research, whether primary or secondary analysis work is needed. We will caveat all summary statements and recommendations with the limitations of the methodology but treat this as a necessary step in addressing the current state of the CBT evidence base.

The mapping exercise will identify in which health problems, across which sub-groups, contexts and with what format, CBT has been evaluated, thereby identifying gaps which have not been examined with a high quality systematic review.

The synthesis stage can identify if CBT can produce long term changes in quality of life. It will also present, with varying degrees of confidence, where CBT does or does not produce generic or problem-specific long term changes upon specific functions.

We will search Prospero, ClinicalTrials.gov and ICTRP to identify on-going, completed or published trials or systematic reviews which have addressed the areas we recommend for further research. This summary will lead to a set of recommendations regarding the prioritisation of primary or secondary research into areas where we cannot generalise the clinical effectiveness findings and the evidence base is weak.

Dissemination plan

An overview of the project will be published in the NIHR journals library. We plan to prepare secondary publications detailing the generic effects of CBT upon HRQL, depression, anxiety, psychosis and the most commonly found physical/physiological outcome. When there is sufficient data we will publish health problem specific overview papers. We hope to present the findings at international conferences to make sure the information is communicated to the patient population perhaps via patient conferences and/or social media.

For peer review only

References

1. Grazebrook KaG, A. What are cognitive and/or behavioural psychotherapies. UKCP/BACP Mapping psychotherapy exercise. . 2005.
2. Mansell W. The seven C's of CBT: A consideration of the future challenges for cognitive behaviour therapy. *Behavioural and Cognitive Psychotherapy.* 2008;.36(6):pp.
3. Roth A, Pilling S. *The competencies required to deliver effective Cognitive and Behavioural Therapy for people with Depression and Anxiety disorders. .* London: Department of Health.; 2007.
4. Lockwood C, Page T, Conroy-Hiller T. Effectiveness of individual therapy and group therapy in the treatment of schizophrenia. *International Journal of Evidence-Based Healthcare.* 2004;.2(10):pp.
5. Richmond H, Hall AM, Copsey B, et al. The Effectiveness of Cognitive Behavioural Treatment for Non-Specific Low Back Pain: A Systematic Review and Meta-Analysis. *Plos One.* 2015;10(8).
6. Ballesio A, Aquino MRJV, Feige B, et al. The effectiveness of behavioural and cognitive behavioural therapies for insomnia on depressive and fatigue symptoms: A systematic review and network meta-analysis. *Sleep Medicine Reviews.* 2018:114-129.
7. Brasure M, Fuchs E, MacDonald R, et al. Psychological and Behavioral Interventions for Managing Insomnia Disorder: An Evidence Report for a Clinical Practice Guideline by the American College of Physicians. *Ann Intern Med.* 2016;165(2):113-124.
8. Johnson JA, Rash JA, Campbell TS, et al. A systematic review and meta-analysis of randomized controlled trials of cognitive behavior therapy for insomnia (CBT-I) in cancer survivors. *Sleep Med Rev.* 2016;27:20-28.
9. Burns AM, Erickson DH, Brenner CA. Cognitive-behavioral therapy for medication-resistant psychosis: a meta-analytic review. *Psychiatr Serv.* 2014;65(7):874-880.
10. Cormac I, Jones C, Campbell C. Cognitive behaviour therapy for schizophrenia. *The Cochrane database of systematic reviews.* 2002(1):CD000524.
11. Durham RC CJ, Power KG, Sharp DM, Macdonald RR, Major KA, et al. Long-term outcome of cognitive behaviour therapy clinical trials in central Scotland.

. *Health Technol Assess.*9(42).
12. Perlis RH. Cognitive behavioural therapy has short term but not long term benefits in people with residual symptoms of depression. *Evidence-based mental health.* 2005;8(3):75.
13. CRD. *Undertaking Systematic Reviews of Research on Effectiveness: CRD's Guidance for Carrying Out or Commissioning Reviews (CRD Report 4) 2nd edn. .* York: NHS Centre for Reviews and Dissemination, University of York.; 2001.
14. Higgins JP, Green S. *Cochrane handbook for systematic reviews of interventions.* Vol 5: Wiley Online Library; 2008.
15. Cochrane. The Cochrane Comparing Multiple Interventions Group

Accessed 6th Jan 2016.
16. ISSG. Filters to Identify Systematic Reviews. https://sites.google.com/a/york.ac.uk/issg-search-filters-resource/filters-to-identify-systematic-reviews. Accessed 3 August, 2016.
17. University M. Search Strategies for PsycINFO in Ovid Syntax 2016; https://hiru.mcmaster.ca/hiru/HIRU_Hedges_PsycINFO_Strategies.aspx. Accessed 10 January 2018.
18. Grossman PBaH, J N. Self-control interventions with internalizing disorders: A review and analysis. *School Psychology Review.* 1992;21(2):229-245.
19. Covidence. Covidence: Faster systematic reviews. https://www.covidence.org/. Accessed June, 2016.
20. Moher D, Liberati A, Tetzlaff J, Altman DG, Grp P. Preferred Reporting Items for Systematic Reviews and Meta-Analyses: The PRISMA Statement. *Plos Med.* 2009;6(7).

21. Shea BJ, Reeves BC, Wells G, et al. AMSTAR 2: a critical appraisal tool for systematic reviews that include randomised or non-randomised studies of healthcare interventions, or both. *BMJ.* 2017;358:j4008.

22. Hempel S, Shekelle PG, Taylor SL, et al. Evidence map of mindfulness. *VA-ESP Project #05-226.* 2014.

23. Shea R, Wells, Thuku, Hamel. AMSTAR 2: a critical appraisal tool for systematic reviews that include randomised or non-randomised studies of healthcare interventions, or both. *BMJ.* 2017.

For peer review only

Appendix A: Search Strategy for MEDLINE

Database & platform: Ovid MEDLINE(R) Epub Ahead of Print, In-Process & Other Non-Indexed Citations, Ovid MEDLINE(R) Daily and Ovid MEDLINE(R) 1946 to Present

Search strategy development date: 9 March 2018

1. (cognitive adj2 behavio?r adj3 (therap$ or theor$ or intervention$ or train$ or treatment$ or psychotherap$ or programme$ or program$ or method$ or approach$)).ti,ab,kw.

2. (cognitive adj2 behavio?ral adj3 (therap$ or theor$ or intervention$ or train$ or treatment$ or psychotherap$ or programme$ or program$ or method$ or approach$)).ti,ab,kw.

3. CBT.ti,ab,kw.

4. Cognitive Therapy/

5. or/1-4

6. Meta-Analysis as Topic/

7. meta analy$.tw.

8. metaanaly$.tw.

9. Meta-Analysis/

10. (systematic adj (review$1 or overview$1)).tw.

11. exp Review Literature as Topic/

12 or/6-11

13. cochrane.ab.

14. embase.ab.

15. (psychlit or psyclit).ab.

16. (psychinfo or psycinfo).ab.

17. (cinahl or cinhal).ab.

18. science citation index.ab.

19. bids.ab.

20. cancerlit.ab.

21. or/13-20

22. reference list$.ab.

23. bibliograph$.ab.

24. hand-search$.ab.

25. relevant journals.ab.

26. manual search$.ab.

27. or/22-26

28. selection criteria.ab.

For peer review only

29. data extraction.ab.

30. 28 or 29

31. Review/

32. 30 and 31

33. Comment/

34. Letter/

35. Editorial/

36. animal/

37. human/

38. 36 and 37

39. 36 not 38

40. or/33-35,39

41. 12 or 21 or 27 or 32

42. 41 not 40

43. 5 and 42

44. limit 43 to yr="1992-2018"

For peer review only

PRISMA-P 2015 Checklist

This checklist has been adapted for use with protocol submissions to *Systematic Reviews* from Table 3 in Moher D et al: Preferred reporting items for systematic review and meta-analysis protocols (PRISMA-P) 2015 statement. *Systematic Reviews* 2015 **4**:1

Section/topic	#	Checklist item	Information reported: Yes	Information reported: No	Line number(s)
**ADMINISTRATIVE INFORMATION**					
**Title**					
Identification	1a	Identify the report as a protocol of a systematic review	☒	☐	1
Update	1b	If the protocol is for an update of a previous systematic review, identify as such	☐	☐	Not applicable
**Registration**	2	If registered, provide the name of the registry (e.g., PROSPERO) and registration number in the Abstract	☒	☐	64-66
**Authors**					
Contact	3a	Provide name, institutional affiliation, and e-mail address of all protocol authors; provide physical mailing address of corresponding author	☒	☐	4-21
Contributions	3b	Describe contributions of protocol authors and identify the guarantor of the review	☒	☐	23-27
**Amendments**	4	If the protocol represents an amendment of a previously completed or published protocol, identify as such and list changes; otherwise, state plan for documenting important protocol amendments	☐	☐	Not applicable
**Support**					
Sources	5a	Indicate sources of financial or other support for the review	☒	☐	37-41
Sponsor	5b	Provide name for the review funder and/or sponsor	☒	☐	29-35
Role of sponsor/funder	5c	Describe roles of funder(s), sponsor(s), and/or institution(s), if any, in developing the protocol	☒	☐	34-35
**INTRODUCTION**					
**Rationale**	6	Describe the rationale for the review in the context of what is already known	☒	☐	112-118
**Objectives**	7	Provide an explicit statement of the question(s) the review will address with reference to	☒	☐	107-110 & 119-128

BioMed Central
The Open Access Publisher

For peer review only - http://bmjopen.bmj.com/site/about/guidelines.xhtml

2

Section/topic	#	Checklist item	Information reported Yes	Information reported No	Line number(s)
		participants, interventions, comparators, and outcomes (PICO)			
**METHODS**					
**Eligibility criteria**	8	Specify the study characteristics (e.g., PICO, study design, setting, time frame) and report characteristics (e.g., years considered, language, publication status) to be used as criteria for eligibility for the review	☒	☐	145-181
**Information sources**	9	Describe all intended information sources (e.g., electronic databases, contact with study authors, trial registers, or other grey literature sources) with planned dates of coverage	☒	☐	182-206
**Search strategy**	10	Present draft of search strategy to be used for at least one electronic database, including planned limits, such that it could be repeated	☒	☐	Appendix A
STUDY RECORDS					
Data management	11a	Describe the mechanism(s) that will be used to manage records and data throughout the review	☒	☐	207-211
Selection process	11b	State the process that will be used for selecting studies (e.g., two independent reviewers) through each phase of the review (i.e., screening, eligibility, and inclusion in meta-analysis)	☒	☐	212-225
Data collection process	11c	Describe planned method of extracting data from reports (e.g., piloting forms, done independently, in duplicate), any processes for obtaining and confirming data from investigators	☒	☐	226-230
**Data items**	12	List and define all variables for which data will be sought (e.g., PICO items, funding sources), any pre-planned data assumptions and simplifications	☒	☐	231-242
**Outcomes and prioritization**	13	List and define all outcomes for which data will be sought, including prioritization of main and additional outcomes, with rationale	☒	☐	278-299
**Risk of bias in individual studies**	14	Describe anticipated methods for assessing risk of bias of individual studies, including whether this will be done at the outcome or study level, or both; state how this information will be used in data synthesis	☒	☐	243-251
DATA					
**Synthesis**	15a	Describe criteria under which study data will be quantitatively synthesized	☒	☐	325-337
	15b	If data are appropriate for quantitative synthesis, describe planned summary measures, methods of handling data, and methods of combining data from studies, including any planned exploration of consistency (e.g., I^2, Kendall's tau)	☒	☐	300-323
	15c	Describe any proposed additional analyses (e.g., sensitivity or subgroup analyses, meta-	☒	☐	339-348

BioMed Central
The Open Access Publisher

For peer review only

Section/topic	#	Checklist item	Information reported Yes	Information reported No	Line number(s)
		regression)			
	15d	If quantitative synthesis is not appropriate, describe the type of summary planned	☒	☐	253-271
**Meta-bias(es)**	16	Specify any planned assessment of meta-bias(es) (e.g., publication bias across studies, selective reporting within studies)	☒	☐	349-353
**Confidence in cumulative evidence**	17	Describe how the strength of the body of evidence will be assessed (e.g., GRADE)	☒	☐	316-323

For peer review only

BioMed Central
The Open Access Publisher
